# Human induced pluripotent stem cells (hiPSC), enveloped in elastin-like recombinamers for cell therapy of type 1 diabetes mellitus (T1D): preliminary data

**DOI:** 10.3389/fbioe.2023.1046206

**Published:** 2023-04-25

**Authors:** Pia Montanucci, Teresa Pescara, Alessia Greco, Giuseppe Basta, Riccardo Calafiore

**Affiliations:** Division of Internal Medicine and Endocrine and Metabolic Sciences (MISEM), Laboratory for Endocrine Cell Transplants and Biohybrid Organs, Department of Medicine and Surgery, University of Perugia, Perugia, Italy

**Keywords:** T1D, human induced pluripotent stem cells, elastin-like recombinamers, insulin, cell therapy

## Abstract

**Introduction:** Therapeutic application and study of type 1 diabetes disease could benefit from the use of functional β islet-like cells derived from human induced pluripotent stem cells (hiPSCs). Considerable efforts have been made to develop increasingly effective hiPSC differentiation protocols, although critical issues related to cost, the percentage of differentiated cells that are obtained, and reproducibility remain open. In addition, transplantation of hiPSC would require immunoprotection within encapsulation devices, to make the construct invisible to the host’s immune system and consequently avoid the recipient’s general pharmacologic immunosuppression.

**Methods:** For this work, a microencapsulation system based on the use of “human elastin-like recombinamers” (ELRs) was tested to envelop hiPSC. Special attention was devoted to *in vitro* and *in vivo* characterization of the hiPSCs upon coating with ERLs.

**Results and Discussion:** We observed that ELRs coating did not interfere with viability and function and other biological properties of differentiated hiPSCs, while *in vivo*, ELRs seemed to afford immunoprotection to the cell grafts in preliminary *in vivo* study. The construct ability to correct hyperglycemia *in vivo* is in actual progress.

## 1 Introduction

The loss of insulin-producing pancreatic β cells will reach approximately either 70%–100% in type 1 diabetes (T1D), due to islet β cell directed autoimmunity, or up to 65% in type 2 diabetes (T2D), depending upon miscellaneous factors, such as glucotoxicity, amyloid deposits and inflammation (Butler et al., 2003; Donath et al., 2008; Weir and Bonner-Weir, 2013). Hence, for both diabetes types, but especially for T1D, β cell replacement therapy may be regarded as a potential cure. There are a few cellular types that could serve for β cell surrogates for substitution purposes and in particular, human embryonic stem cells (hESC) (Van Hoof et al., 2009) and human induced pluripotent stem cells (hiPSC) (Pagliuca et al., 2014). Both cell types may virtually be suitable for differentiation into pancreatic functionally competent β-like cells. But unfortunately, use of hESC remains ethically controversial. Therefore, the landmark discovery of hiPSC (Takahashi and Yamanaka, 2006), with remarkable similarities to hESC, and relatively easy to derive from somatic cells/tissues, may fuel hopes towards validation of a suitable cell model for replacement of the original β cells. Differently from hESC, hiPSC are derived by “reprogramming” somatic cells into a pluripotent state by over-expression of a key set of transcriptional factors (Borgohain et al., 2019).

This approach could allow for development of patient-tailored hiPSC that are theoretically protected from immune rejection, and could be safely re-implanted, but also for setting novel approaches for heritable genetic disorders in their human cell types (Maxwell and Millman, 2021). Nevertheless, substantial generation of mature pancreatic β-like cells with a complete array of functional properties continues to be a work in progress (Agrawal et al., 2021).

To spare the recipients’ chronic pharmacological immunosuppression, graft immunoprotection within “classic” highly biocompatible sodium alginate-based microcapsules can represent a valid alternative as demonstrated by our previous pilot clinical trials (Basta et al., 2011). However, the average microcapsules diameter of about 500 μm limits their use when a large volume of tissue needs to be transplanted or re-transplanted. An interesting alternative is represented by the emerging new polymer technologies consisting of recombinamers (Rodriguez-Cabello et al., 2011a). Chemically cross-linked hydrogels capsules and cell coatings, based on human elastin-like recombinamers (ELRs) consist of peptide materials obtained as recombinant proteins from a purely synthetic DNA constructs, upon tailored engineering design of the final material encoded composition (Rodriguez-Cabello et al., 2011b; Ibanez-Fonseca et al., 2019). ELRs consisting of repeating sequences of Elastin-like polypeptide Val-Pro-Gly-Val-Gly (VPGVG) found in the mammalian elastin, have been intensively studied in the last few years under several aspects, in an attempt to mimic biology and physical chemistry of the normal extracellular matrix (ECM) (Miao et al., 2003; Lee et al., 2013). Elastin is one of the main components of the ECM, and it is deemed to confer elasticity to a variety of organs and tissues, such as lungs, skin and blood vessels, among others, while also contributing on cell signaling (Yeo et al., 2015; Mecham, 2018). Formed by the lysis oxidase-mediated cross-linking of lysine residues present in its soluble precursor, tropoelastin, it is composed of highly repetitive and well-conserved domains, including the hydrophobic VPGVG pentapeptide, first described by Gray et al. (Gray et al., 1973). However, one of the major limitations at this point was that these primitive elastin-like (poly) pentapeptides (ELPs) were chemically synthesized, hindering the achievement of long polypeptides (Nilsson et al., 2005; Borra and Camarero, 2013). This issue was addressed with the advent of the recombinant DNA technology which was easily adapted to the expression of structural protein polymers in heterologous hosts, mainly *Escherichia coli* (McPherson et al., 1992). This also increased the versatility of the ELPs, and allowed for the combination of different structural protein domains, or the inclusion of bioactive amino acid sequences (Nicol et al., 1992). Furthermore, the polymeric and recombinant nature of these ELPs led to a new nomenclature proposed by Rodríguez-Cabello: “elastin-like recombinamers (ELRs),” in order to recapitulate both features in a single term (Rodriguez-Cabello et al., 2009). ELRs are biocompatible: the immune system just ignores these polymers that are undistinguishable from native elastin.

Coatings made of ELRs are semipermeable and permselective, which means that the membrane will allow oxygen, nutrients, and glucose inflow and insulin outflow, while preventing access to both humoral and cellular mediators of the host’s immune system.

We aimed at incorporating β-like cell spheroids made of hiPSC within ELRs-based micro-coatings, to create physical and biocompatible polymer barriers that are ultimately able to protect hiPSC from the host’s immune response upon transplantation, with no adverse effects on their viability and functional properties.

## 2 Materials and methods

### 2.1 hiPSC procurement and culture

hiPSC were purchased frozen from Alstem Inc. Upon thawing, the cells were cultured on 6 cm dishes coated with Vitronectin XF™ (Stemcell Technologies Inc.), and NutriStem XF™ (Biological Industries) medium. Undifferentiated cells were culture maintained by passaging the cell clusters upon treatment with 0.5 mM EDTA. Freezing was performed by EZStem freezing medium™ (Alstem Inc.) as recommended by the manufacturer, while thawing was performed in NutriStem XF™ medium with 10 μM ROCK inhibitor (Sigma-Aldrich).

### 2.2 hiPSC β-like cell differentiation

To create hiPSC spheroids for subsequent differentiation into β-like cells, using scalable three-dimensional cultures, we adopted a method described by Schulz (Schulz et al., 2012). This is based on use of low adherence 6-well-plates that are kept under constant mechanical agitation (100 rpm) throughout the culture time and the differentiation process. To activate spheroid formation, cells were incubated at a density of 2 × 10^6^ cells/ml in NutriStem XF™ medium supplemented with 10 μM ROCK inhibitor under agitation. Cell differentiation started when the cell clusters ranged on 150–220 μm in diameter, after 48–72 h of incubation. Differentiation of hiPSCs into β cell-like spheroids was performed according to Millman protocol (Millman et al., 2016) (Supplementary Data). The only change was variation of retinoic acid concentration from 2 to 3 μΜ at Stage 3.

### 2.3 Polymer coating of cell clusters

ELRs, for cell encapsulation, were provided by Prof J.C. Rodríguez Cabello, Bioforge Lab, University of Valladolid, Spain (Girotti et al., 2004; Gonzalez de Torre et al., 2014). The two used ELRs were: VKV-cyclo and RGD-N3 (Huising et al., 2018; Docherty and Sussel, 2021).

#### 2.3.1 Coating protocol for hiPSC spheroids

Cell spheroids (∼1,000) were placed in cell strainers measuring of 100 µm diameter. The cell strainer was sequentially dipped in ELRs, starting with RGD-N_3_ and continuing with VKV-cyclo and repeating the process three times. For the first layer with RGD-N_3_, spheroids were incubated for 20 min. The remaining layers were incubated for 5 min each. A total of 6 bilayers (BL) were obtained. The hydrogel formation reaction is a cyclo-addition 1,3-dipolar reaction named “click-chemistry” that takes place *in situ*, under physiological conditions, with high reaction kinetics, in the absence of catalysts, chemical cross-linkers and/or organic solvents, with no generation of toxic sub-products (Testera et al., 2015).

### 2.4 Morphologic, functional and molecular characterization of cell spheroids

#### 2.4.1 Viability assay

Our differentiated spheroids were tested for viability by ethidium bromide (0.2 mg/ml) and fluorescein diacetate (5 mg/ml) staining (Sigma-Aldrich), incubation time 5 min at 37°C, under fluorescence microscopy, using appropriate filter sets. Green signal (fluorescein) evidences live cells; red signal (ethidium bromide) marks dead cells, indicating membranes ruptures thereby binding to nuclear DNA. Cell monolayers viability was examined by phase contrast microscopy.

#### 2.4.2 Immunocytochemistry

Immunocytochemistry was performed on cells seeded on glass slides (12 mm in diameter), or on spheroids treated with 3.7% para-formaldehyde (PFA). Permeabilization (when necessary) with 0.01% triton X-100 in D-PBS and block with 1% BSA in D-PBS for 1 h were performed. Primary antibody was added overnight; thereafter, a secondary antibody was added for 1 h (see supplementary Data). Slides were mounted in SlowFade Gold antifade reagent with DAPI following manufactures’ recommendations (Invitrogen). Images were collected on Nikon Eclipse Ci fluorescence microscope.

#### 2.4.3 Insulin staining

A diphenylthiocarbazone (DTZ) solution (0.250 mg/ml in HBSS) was added to the sample and allowed to stain for 1–2 min at room temperature. This reagent binds to ZN of the insulin molecule evidencing it in red. Upon examination under light microscopy, cell spheroids containing insulin would appear in red.

#### 2.4.4 Histology

For histologic purpose, 3–4 μm thick paraffin-embedded sections of the spheroids underwent staining with hematoxylin/eosin following standard methods.

#### 2.4.5 Transcriptional expression analysis by quantitative PCR

Total cellular RNA was extracted using Direct-zol (Zymo Research). cDNA was synthesized using the iScript cDNA Synthesis Kit (Bio-Rad Laboratories) and was used as a template for quantitative PCR (qPCR). qPCR amplifications were performed using the SsoAdvanced universal SYBR Green Supermix (Bio-Rad Laboratories) and Agilent AriaMx (Stratagene) (Supplementary Data). PCR products showed to be a single PCR product by melting curve and electrophoresis analysis. The relative quantification was determined by the comparative 2^−ΔΔCT^ method using GAPDH as reference gene.

#### 2.4.6 Insulin secretion measurement

Insulin secretion was measured at final 7 days of cell differentiation (medium 6) that was insulin-free, upon static incubation with glucose: spheroids (50 spheroids/well, in triplicate) were pre-incubated for 1 h in pH 7.4 KREBS buffer, then in the same buffer supplemented with glucose. Two-hour stepwise standard static incubation with sequential 2 and 20 mM glucose for three times was performed. Insulin was extracted upon overnight incubation with 30 mM KCl. Secreted insulin content was assayed by RIA (Bouty s.p.a.) and expressed as μΙU/µgDNA.

#### 2.4.7 Transmission electron microscopy

Samples were pre-fixed in 2% glutaraldehyde buffered with 0.2 M Na cacodylate, pH 7.4, for 2 h at 4 C, rinsed in the same buffer, postfixed with 2% osmium tetroxide, in the same buffer for 2 h, dehydrated in ethanol graded series, and embedded in eponaraldite. Ultra-thin sections were stained with uranyl acetate and lead citrate and examined under transmission electron microscopy (TEM) 400 T-Philips at 60 kV.

#### 2.4.8 Cytofluorimetric assay

Cytofluorimetric assessment was performed using fluorochrome-conjugated monoclonal antibodies against Oct4 (PE), Sox2 (PerCy5.5), and Nanog (AlexaFluor488) and relative isotype controls. All antibodies were provided by Thermo Fisher Scientific. Before intracellular staining, the cells were fixed with 0.05% formaldehyde and permeabilized with 0.1% saponin. At least 20,000 events were acquired for each analysis.

### 2.5 *In vivo* studies

#### 2.5.1 Transplant procedure

The transplantation procedure consisted of resuspending approximately 1,000 spheroids in saline and injecting them into the mouse peritoneal cavity. The animals were maintained under pharmacological sedation throughout the procedure. All the *in vivo* studies complied with National and University of Perugia Animal Care and Use Committee guidelines (512/2015 PR).

#### 2.5.2 Explantation

Upon animal sacrifice, the spheroids were retrieved by peritoneal lavage with saline, placed in sterile tubes, and incubated in culture flasks, at 37 C, 95% CO_2_ for further analysis. After 24 h, spheroid aliquots were tested for viability, while the remainder were fixed for histologic assessment and TEM.

### 2.6 Data analysis

All *in vitro* determinations were expressed as mean ± SD from at least three independent experiments and considered significant for *p* values < 0.05. All data analysis was performed using IBM-SPSS version 20.0. We used the *t*-test to compare in the Static Incubation the 2 and 20 mM of each induction and the one-way Anova test to compare KCl treatment with all other conditions. A one-way ANOVA test was used in the qPCR plots to compare our experimental points with whole islets.

## 3 Results

### 3.1 *In vitro* function of differentiated hiPSC

**FIGURE 1:**
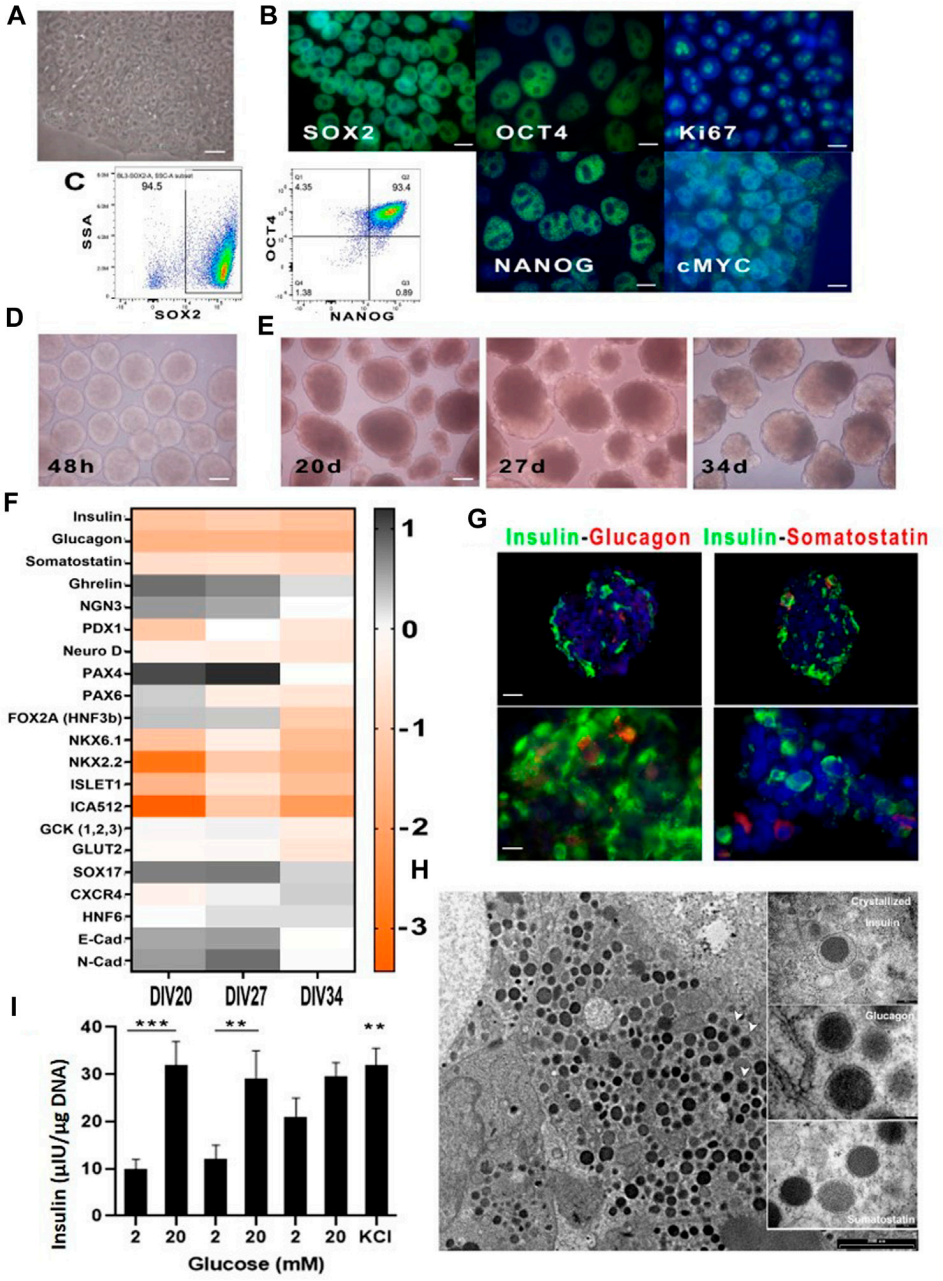
Cultured hiPSC in Nutristem medium before and after differentiation. **(A)** hiPSC cultured on vitronectin XFTM and NutriStem XFTM before differentiation (40X); **(B)** Immunofluorescence analysis for the indicated stem cell markers (100X); **(C)** Representative cytofluorimetric dot plots showing triple positivity to Sox2, Oct4 and Nanog over 90% of basal hiPSC; **(D)** Spheroids after 48 h of mechanical aggregation just before differentiation initiation (4X); **(E)** Spheroids’ morphology at time three of terminal differentiation (10X); **(F)** qPCR for indicated messengers at time three of terminal differentiation; **(G)** Immunofluorescence analysis of cell spheroids at Div27 for insulin-glucagon and insulin-somatostatin: presence of single hormone producing cells was evident (20X, 100X); **(H)** TEM micrographs showing insulin granuli in the cell cytoplasm; white arrows indicate vesicles with crystallized insulin inside them. Photographs in the boxes on the right show enlargements of hormone vesicles found in other spheroids’ cells; **(I)** Static incubation performed on cell spheroids at Div27, was associated with near physiological insulin secretory patterns in response to glucose; also evident was the presence of residual insulin after KCl addition indicating correct cell differentiation. ***p < 0.0001 comparison of 2 mM and 20 mM first stimulation **p < 0.005 for comparison of second stimulation. n.s not significant for third stimulation. KCl significant with *p* < 0.005 in the comparison of 2 mM of the first and second induction.

Under the above indicated culture conditions, cells grew in well-cond clusters with identical morphology (Figure 1A). Immunofluorescence for pluripotency markers confirmed the presence of Oct4, Sox2, Nanog, c-Myc and Ki67 on all cells of each cluster (Figure 1B) and cytofluorimetry confirmed that on average over 90% of cells were triple positive for Sox2, Oct4 and Nanog (Figure 1C).

The selected differentiation protocol (Millman et al., 2016) for turning hiPSCs into insulin-secreting cells performed efficiently. It started at 48 h of the cell aggregation induction on a rotating plate. Figure 1D shows morphology of the cell aggregates just before the differentiation onset and thereafter at day 20 that represented the starting day of stage 6 of terminal differentiation, then at day 27 and 34, namely, the two selected times for differentiation ending (Figure 1E). Morphology changes of the aggregates were clear, as they increased in diameter and were no longer homogeneous as compared to 48 h earlier.

qPCR (Figure 1F) for the various factors involved in the differentiation process (namely, PDX-1, NKX6.1, SOX9, PTF1a, FOXA2, SOX17, CXCR4, c-KIT, Glut2 and Glucokinase), other than those expressed by β cells (MafA, MAfB, NKX2.2 and NKX6.1) showed modulation of most of them at day 27 of maturation. All spheroids that reached day 27 were then characterized, in more details, looking for content of the three major pancreatic hormones (Figure 1G). In fact, fluorescence microscopy confirmed that insulin was very well represented, while glucagon and somatostatin were also detectable but at lower intensity. Confirmation of insulin production by the differentiated cell spheroids was obtained by TEM (Figure 1H). In fact, we were able to visualize insulin granules, within the cell spheroids, some of which associated with crystallized or recently synthesized insulin.

Some areas of the cell aggregates showed null granule content, likely following a divergent differentiation pathway, i.e., towards a liver cell-type phenotype. This is plausible given ontogenetic proximity of the pancreas and liver. At functional level, static incubation with glucose at different concentration (Figure 1I) confirmed that most batches of correctly differentiated hiPSCs were able to respond physiologically to glucose concentration shifts. In these batches, the amount of secreted insulin was low, as compared to human islets, but the secretory kinetics looked normal. Final insulin output at 30 mM KCl confirmed remainder insulin, at the end of the test, indicating that β-like cells were able to store insulin. Physiological insulin secretory kinetics is supported by TEM imaging that clearly shows insulin containing cytoplasmic granules (Figure 1H). Nevertheless, in some batches, where differentiation was sub-optimal, the amount of insulin secreted, as compared to human islets, was very low and the kinetics was abnormal, as already reported in the literature (Docherty and Sussel, 2021).

### 3.2 ELRs coating of cell spheroids

**FIGURE 2:**
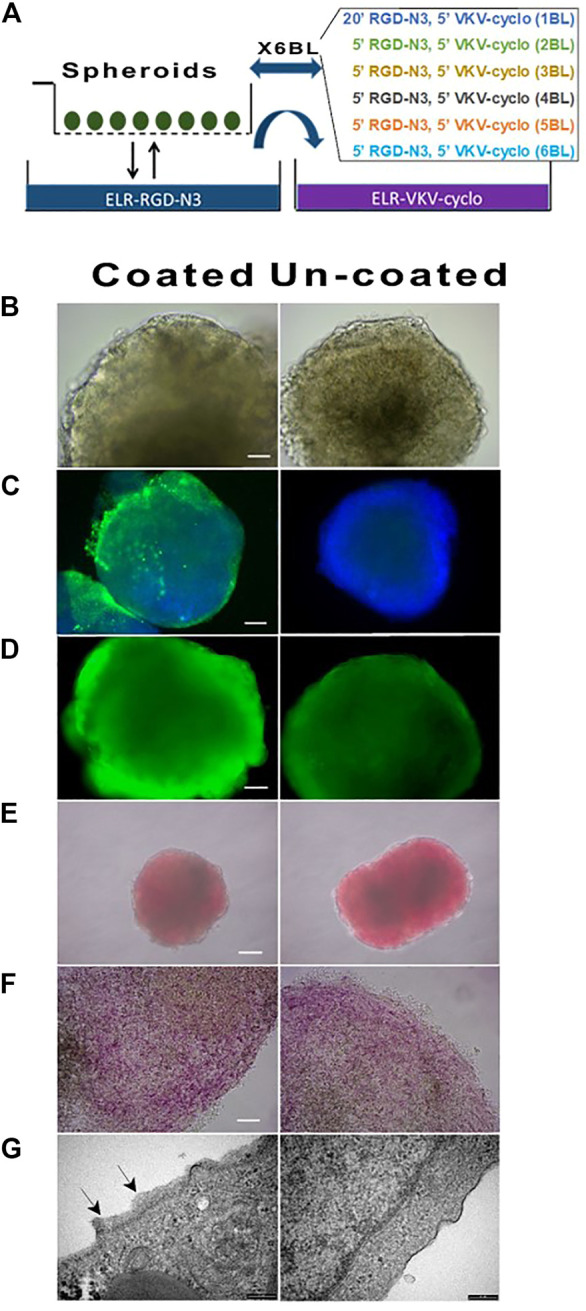
Morphology, viability, and DTZ assay of coated and un-coated hiPSC at DIV27. **(A)** Schematic representation of spheroids coating procedures. **(B)** Morphology (40X); **(C)** Immunofluorescence performed with anti-elastin antibody: the thin layer of ELRs on coated unlike uncoated cell spheroids was evident (20X); **(D)** Viability (40X) and **(E)** DTZ staining of coated vs un-coated spheroids did not show differences (4X); **(F)** Details of spheroids DTZ-stained upon squeezing by coverslip: individual areas within the cell spheroids intensely stained in red were visible (40X); **(G)** TEM micrograph showed coating of ELRs (black arrows) as compared to untreated cells spheroids.

The coating protocol consisted of 6 layers RDG-N3/VKV-Cyclo and was performed as indicated in the Materials and Methods on differentiated cell spheroids (Figure 2A). At the end of procedure, we found no differences in terms of either morphology (Figure 2B), or viability (Figure 2D) (always greater than 95%), or hormones content (Figures 2E, F), or messengers, or functional performance (data not shown). The immunofluorescence performed with an anti-elastin antibody (Figure 2C) showed a specific signal on the aggregates, although in this instance, cryostat slices was not associated with full visualization of the coating on the spheroids. Moreover, we observed that ELR-coated spheroids, culture maintained *in vitro*, did not show any kind of changes, with the rotation plate switched off to protect coating. The coated spheroids could be safely moved, as documented by transfers from a lab to another, within the EU Horizon 2020 Consortium. We observed, however, that the coating was very vulnerable, in the initial but not advanced stages of polymerization, to working temperature, that required special attention. All batches of differentiated spheroids were tested with DTZ (Figure 2E) to detect insulin, in the possibly differentiated β cells. We confirmed that the obtained aggregates were intensely and homogeneously stained in red, demonstrating the presence of the processed and zinc-conjugated insulin molecules. Figure 2 shows the same cell aggregates red stained with DTZ before (Figure 2E) and after disruption by a coverslip (Figure 2F), containing insulin. TEM examination (Figure 2G) of the surface of coated vs uncoated spheroids showed the presence of a surface layer whose thickness ranged on 20 nm–200 nm.

### 3.3 Identification of cell markers

#### 3.3.1 UCN3 and HLAG5

**FIGURE 3:**
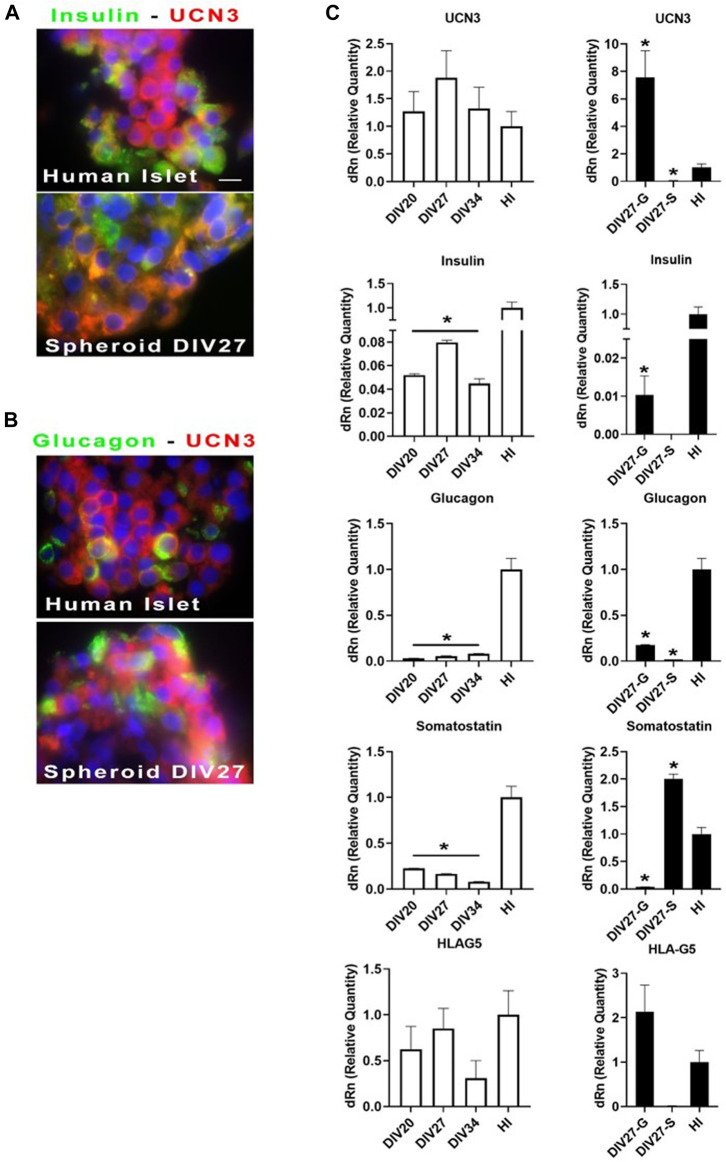
**(A, B)** Immunofluorescence analysis of cell spheroids at Div27 for the indicated antibodies in comparison with human islets (100X). Orange merging color (between green and red) might suggest although not prove that UCN3 co-localize with insulin and glucagon. **(C)** qPCR for the indicated messengers: white histograms showed relative quantity of the selected messengers for the three times of terminal differentiation stages as compared with whole human islets. Div27 looked the best differentiation time; black histograms showed that differentiation at Div27 was more oriented toward glucagon (Div27-G) or somatostatin (Div27-S) production in comparison with human islets *p < 0.05.

For cell identity marker throughout analysis by immunofluorescence and qPCR, we included Urocortin 3 (UCN3). In fact, its expression appears at late stages of β/α-cell differentiation, and robustly increases upon differentiation into mature endocrine cells (Li et al., 2003; Huising et al., 2018). Figure 3 shows images of differentiated cell aggregates in comparison with mature human islets. Staining for UCN3 within insulin (Figure 3A) and glucagon (Figure 3B) positive cells are shown. Images, acquired by immunofluorescence microscope, showed that UCN3-expressing cells exhibited a merging color between red and green: this may suggest, although not prove, a potential co-localization with insulin and glucagon. HLA-G5 behad exactly like UCN3, in terms of immunostaining results (data not show). We then assessed the levels of UCN3 mRNA in differentiated cell spheroids, in comparison with mature human islets (Figure 3C). White histograms showed optimal differentiation: in this instance, relative levels of UCN3 mRNA moved in concert with the levels of insulin mRNA. Furthermore, the expression levels were superimposable to those of human islets. As compared with human islets, messenger levels for insulin were, even in these optimal cases, about 15-fold lower while the levels of somatostatin and glucagon also were much lower than those of islets. HLA-G5 mRNA, present in the insulin secretory granules (Cirulli et al., 2006) like UCN3, is another excellent marker. In our differentiated spheroids, the expression levels of HLA-G5 were comparable to those of human islets and followed as similar patterns as UCN3 in the aggregates, at advanced stages of differentiation. On the contrary, black histograms, at day 27 of differentiation, depicted two representative cases of suboptimal differentiation. At “Div27-G″ there was production of glucagon mRNA, while insulin messenger was 10 times lower in comparison with the best obtained differentiation, and somatostatin mRNA was almost undetectable. On the other hand, at “Div27-S” somatostatin mRNA was well expressed as compared to human islets but no glucagon nor insulin were detected. In these two cases messengers for UCN3 and HLA-G5 were still expressed. Likely, the expression of UCN3 in differentiated hiPSC spheroids does seem to discriminate maturity of the system according to the presence or absence of this marker.

### 3.4 *In vivo* studies

#### 3.4.1 *In vivo* survival study in NOD/SCID mice

**FIGURE 4:**
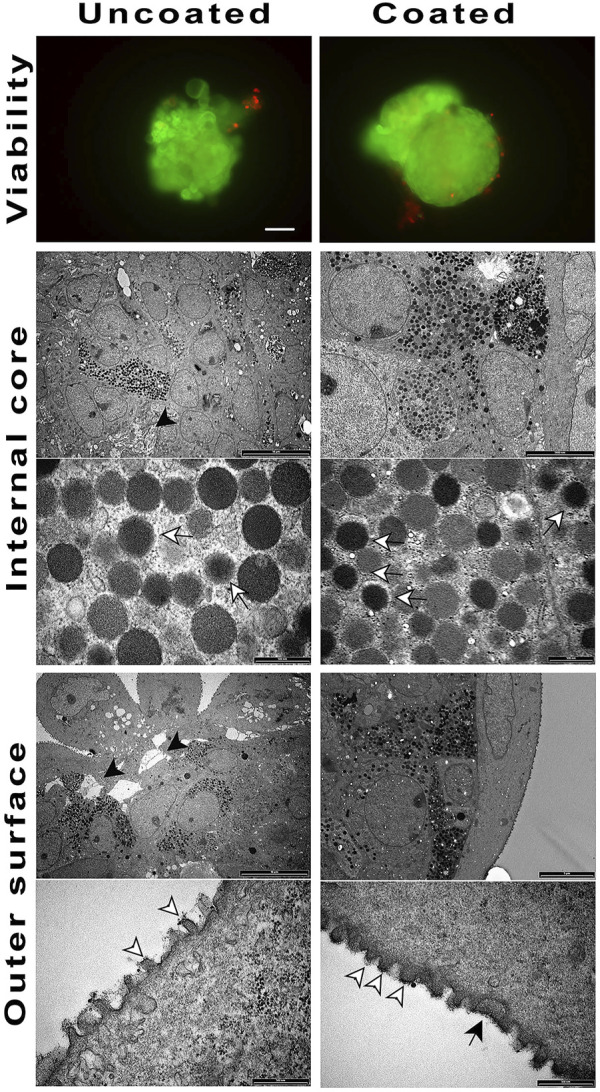
Coated and un-coated cell spheroids transplanted at Div27 in NOD/SCID mice and retrieved 14 days later. Viability was maintained with the un-coated cell spheroids looking inhomogeneous (black arrow heads) as compared to the coated ones (20X). TEM examination of the inner core showed the presence of hormone granules in both samples (white arrows). ELRs coating was still detectable on the outer spheroids surface (black arrow). White arrowheads showed membrane evaginations.

Differentiated spheroids were transplanted into immune-incompetent NOD/SCID mice to determine both, their ability to survive *in vivo*, and the most suitable graft site. Initially, we grafted coated and un-coated spheroids in the mice epididymal fat or intramuscularly; however, no transplanted material could be retrieved from these sites. In effects, the peritoneal cavity resulted the only viable transplant site. In this mouse model, there were slight differences in terms of material survival, although the uncoated, unlike coated spheroids, showed areas of dead cells and slightly lesser morphologic integrity (Figure 4). The surface analysis of the retrieved spheroids showed the presence of the coating, somewhere uneven. Moreover, the surface of the coated, unlike uncoated, spheroids showed innumerable evaginations.

#### 3.4.2 Assessment of *in vivo* survival in CD1 mice

**FIGURE 5:**
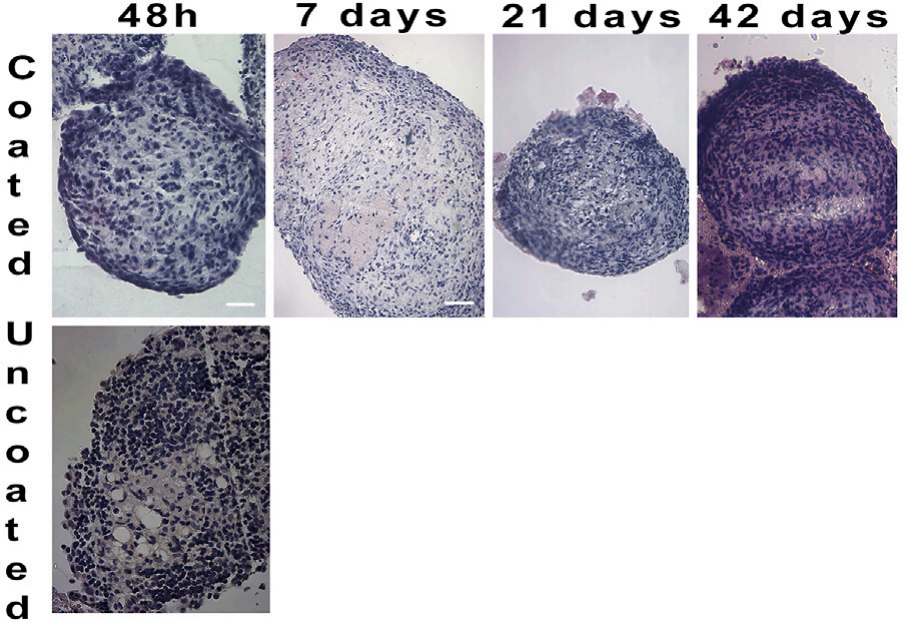
Hematoxylin/Eosin staining on cell spheroids transplanted in CD1 mice and retrieved at different time periods. After 48 h no un-coated spheroids were detected (4X). Coated spheroids were retrieved from CD1 mice throughout 42 days of graft, and histological staining showed well defined and compact cell composition (4X).

To determine the ELR immunobarrier competence we grafted ELR-microencapsulated cell spheroids also into immunocompetent xenogeneic-discordant CD1 mice. This mouse model was selected because of the encouraging data obtained in the NOD/SCID mice, and to prove/unprove ELRs immunoprotective effectiveness in a xeno-discordant graft setting. In the various planned experiments, we had grafted intraperitoneally ∼1,000 both, coated and uncoated spheroids, and scheduled periodic retrievals either in the short- (24–48 h) or in the very long- (42 days) term post-transplant. The experiments provided homogeneous results (Figure 5): at 48 h of graft the uncoated spheroids provoked an immune response in form lymphocytic and macrophagic reaction to the transplanted tissue. These findings were assessed by analyzing, histologically, the few uncoated aggregates that could be recovered. On the contrary, in the instance of ELR-coated spheroids, it was possible to retrieve them at all scheduled times, namely, 48 h, and 7, 21, and 42 days post-graft. Histological examination was consistent with preservation of cell morphology and viability while the amount of produced insulin resulted either increased or unchanged, as compared to the time of transplantation. Gross inspection of the peritoneum showed no signs of inflammation, with the peritoneal mouse cells, collected by lavage, consisting of macrophage and lymphocyte cell populations (Supplementary Data).

## 4 Discussion

Diabetes results from dysfunction or complete destruction of insulin-producing β cells within pancreatic islets of Langerhans. In T1D, the uncontrollable elevation of blood glucose will ineludibly lead to daily exogenous insulin supplementation. The Edmonton protocol has provided evidence in support to application of a cell-based therapy for T1D using donor cadaveric islets under general immunosuppression (Shapiro et al., 2000). The possibility to avoid pharmacological immunosuppression by use of microencapsulation for islet graft immunoprotection, further implemented of this approach (Basta et al., 2011). Nevertheless, widespread success of this cellular therapeutic option for diabetes would require access to unlimited quantities of highly viable and functional β-cells. Moreover, time-related functional decline of the grafted islet function, in conjunction to imperfect means for graft immunoprotection, continue to represent major limitations. On this purpose, substantial progress has been made in the development of stem cell-derived β-like cells using hiPSC (Augsornworawat et al., 2020). However, differentiation of hiPSCs is hampered by several hurdles, which often translates into suboptimal results. During our work, we have experienced the extreme care required to manipulate and differentiate these cells as well as the modest amount of insulin obtained even under the best experimental conditions. These issues were not well defined in the literature until recently, when Bourgeois (Bourgeois et al., 2021) highlighted the specific challenges that are associated with production of mature β cells. Moreover, Millman (Hogrebe et al., 2021) published a detailed protocol on how to obtain an optimal differentiation of hiPSCs focusing on critical steps of the process and also proposing substantial changes, as compared to prior published data. This may revive interest on the potential use of these cells for the therapy of diabetes mellitus.

In this work, efficiently differentiated cell spheroids were over-layered with ELRs and examined *in vitro* and *in vivo*. The analyses showed that the presence of the elastomer did not compromise the acquired cell differentiation and that it was possible to transplant the spheroids and retrieve them at 42 days of graft in immunocompetent mice. In addition to the immune-protective properties, the elastin-based coating has been shown to provide other benefits to encapsulated cell aggregates, especially in terms of stability. The coated vs uncoated cell clusters showed superior integrity, with favorable consequences in terms of their stability over time. Ultimately, the ELRs limited thickness and ability to evenly adhere to the cell aggregates surface, resulted in graft volume containment with relevant benefits.

A key aspect to demonstrate suitability of differentiated cell aggregates for transplantation is demonstration of their ability to survive in the implantation site, namely, the peritoneal cavity. We had explored other sites, namely, muscle and the epidydimal fat pad but we had been unable to detect and retrieve the grafted material. That is why we switched to the peritoneal cavity for graft purposes. Using the immunodeficient NOD/SCID mice, we observed that although both, coated and uncoated cell spheroids, showed satisfactory viability at 14 days after transplantation, viability was higher for coated vs uncoated aggregates. The mechanism that seems to favor a better survival of the aggregates enveloped in elastin could depend on the better stability that the elastin fibers confer to the cell clusters. In fact, a stable cell aggregate has a lower tendency to flake, a phenomenon that negatively affects cell viability, thereby limiting survival of the transplanted tissue. Since the tendency to flake has been observed in uncoated aggregates already *in vitro*, it is likely that this may *a fortiori* occur *in vivo*, given the higher local mechanical stress.

Data obtained in immunocompetent CD1 mice proved to be interesting, especially in terms of responsiveness of the immune system to the grafted material. First, animals transplanted with coated spheroids showed no obvious signs of distress attributable to a hyperacute rejection throughout the experiment. In addition, upon aggregates explant, at inspection, no signs of inflammation of the peritoneal cavity were evident at either short- (48 h) or long- (42 days) term. In the instance of uncoated aggregates, it was possible to recover spheroids only after 48 h post-graft, while at other times no cell clusters were detectable. Moreover, the few retrieved aggregates showed clear signs of lymphocytic and macrophagic infiltration.

Upon termination of the study, we performed peritoneal lavage of the recipient animals with saline to examine the cell populations involved in the graft-directed reaction. In the uncoated spheroids group, we found abundant macrophages and lymphocytes, whereas in the coated spheroids group we found neutrophils and rare lymphocytes. Uncoated spheroids at 7,21 and 42 days of transplant were not detected, while the peritoneal lavage cells of the coated spheroids group showed not specific cell reaction, comprised of few not activated lymphocytes and macrophages. While further study will be necessary to characterize the identified cell populations by flow cytometry, we maintain that the coated spheroids to do not provoke an immune response, but just not specific inflammation.

Despite the excellent results achieved in the short-as well as mid-term, it will be necessary to complete long-term studies to judge about functional performance of the conformal ELRs coatings. In addition, in this work we were able to show that the expression of UCN3 in differentiated hiPSCs discriminated the maturity of this cellular system based on the relative presence/levels of this messenger and related protein, as compared with human islets. Furthermore, we described a close association between the expression of insulin, UCN3, and HLA-G5 in spheroids vs human islets.

UCN3 is a member of the corticotropin-releasing factor (CRF) family that selectively binds the G-protein-coupled receptor-2 (CRFR2) (Lewis et al., 2001). Shortly after its discovery, it was found to be expressed in mature β cells (Li et al., 2003). UCN3 is co-released with insulin under high glucose stimulation, and it also promotes somatostatin secretion from δ-cells, which are the primary cells within the islets to express CRHR2 (van der Meulen et al., 2015). UCN3 by acting through CRFR2 is involved in the local regulation of glucagon and insulin secretion (Li et al., 2003). Van der Maulen (Huising et al., 2018) showed that UCN3 expression is associated with the acquisition of functional maturity of β cells. In the case of cells of human origin, the situation is complicated by the fact that cells expressing glucagon also contain UCN3.

In pancreatic endocrine cells, HLA-G is expressed constitutively at low levels, as β2-microglobulin–free heavy chain, and is mainly intracellular (Cirulli et al., 2006). HLA-G may have specialized in increasing the activation/effector thresholds of T-, NK, and antigen presenting cells for the immune protection of the “semi-allogeneic” fetus, at the maternal/fetal interface. It also hallmarks immunologically protected sites or sites of lymphocyte selection. However, many autoantigens in islet immunity compose the secretory granules (Nepom, 1995; Kent et al., 2005; Nakayama et al., 2005). Thus, it is possible that sites of insulin exocytosis may represent subcellular domains where a high density of potentially immunogenic ligands become exposed. Since the activation of autoreactive T-cells depends upon the surface density of antigen/MHC complexes, this may lead to the activation of low-affinity cytotoxic T-cells. Local clustering of immunoregulatory molecules, such a sHLA-G, at sites of granule exocytosis may prevent this unwanted activation. Hence and apparently, this molecule is expressed by insulin-producing cells, within the same secretion vesicles containing insulin and UCN3, but above all it is present in mature β cells. Therefore, UCN3 and HLAG5 can be used alone or together with other markers to assess beta cell maturity.

In conclusion, we have provided initial evidence that hiPSC might serve for β-cell surrogates, although reproducibility of the results may need improvements. Likewise, we showed that ELRs might constitute a new generation of immunoprotective conformal microcapsules, whose long-term performance *in vivo*, in pre-clinical animal models is under actual study.

## Data Availability

The raw data supporting the conclusions of this article will be made available by the authors, without undue reservation.
