# Supplementary material for: Human induced pluripotent stem cells (hiPSC), enveloped in elastin-like recombinamers for cell therapy of type 1 diabetes mellitus (T1D): preliminary data
Source: Front Bioeng Biotechnol. 2023 Apr 25;11:1046206. doi: 10.3389/fbioe.2023.1046206 (PMC10166868; doi:10.3389/fbioe.2023.1046206)
Supplement: Supplementary file 1 [file DataSheet1.docx]

Human Induced Pluripotent Stem Cells (hiPSC), enveloped in Elastin-like recombinamers for cell therapy of type 1 diabetes mellitus (T1D): preliminary data

**Pia Montanucci, Teresa Pescara, Alessia Greco, Giuseppe Basta and Riccardo Calafiore**

Supplementary Material

# Supplementary: Differentiation Protocol

To start production of cell spheroids, a suspension of hiPSCs, obtained from adherent hiPSC cultures, was incubated at a density of 2x106 cells/ml in NutriStem XF medium supplemented with 10μM ROCK inhibitor Y-27632 (Millipore, SCM075). The initial obtained spheroids were uniform in size and shape, with no evidence of cavitations or cyst-like structures. Differentiation began when the spheroids diameter ranged on 150-220μm, after 48 hours of mechanical aggregation. Differentiation of hiPSC spheroids into islet-like Beta cells followed, in most experiments, the Millman protocol [143].

- Stage 1 (3 days): basal medium S1 + 100 ng/ml Activin A (Peprotech. 120-14E) + 3 μM Chir99021 (Sigma, SML1046) for 1 day. Basal medium S1 + 100 ng/ml Activin A for 2 days.

- Stage 2 (3 days): basal medium S2 + 50 ng/ml KGF (Peprotech; AF-100-19).

- Stage 3 (2 days): basal medium S3 + 50 ng/ml KGF + 200 nM LDN193189 (MilliporeSigma; SML0559) + 500 nM PdBU (MilliporeSigma; 524390) + 3 μM retinoic acid (MilliporeSigma; R2625) + 0.25 μM Sant1 (MilliporeSigma; S4572) + 10 μM Y27632. On the second day of stage 2, LDN193189 factor is removed.

- Stage 4 (4 days): basal medium S3 + 5 ng/mL Activin A + 50 ng/mL KGF + 0.1 µM retinoic acid + 0.25 µM SANT1 + 10 µM Y27632.

- Stage 5 (7 days): basal medium S3 + 10 µM ALK5i II (Millipore, 616452-2MG) + 20 ng/mL β cellulin (Peprotech, 100-50) + 0.1 µM retinoic acid + 0.25 µM SANT1 + 1 µM T3 (Millipore, 64245) + 1 µM XXI (MilliporeSigma; 595790).

- Stage 6 (7-14 days): basal medium S6 + 10 µM ALK5i II + 1 µM T3.

The formulations of basal differentiation medium used were the following:

- Basal medium S1: 500 ml of MCDB 131 (Sigma, SLBW2585) supplemented with 0.72 g glucose (B. Braun), 1.83 g sodium bicarbonate (MilliporeSigma; S3817), 10 g bovine serum albumin (BSA) (Sigma, SCLC3856), 10 µL ITS-X (Invitrogen; 51500056), 5 mL of GlutaMAX (Invitrogen; 35050079), 22 mg of vitamin C (MilliporeSigma; A4544) and 10 mL of penicillin/streptomycin (P/S) solution (Gibco, 15140-122).

- Basal medium S2: 500 mL MCDB 131 supplemented with 0.72 g glucose, 1.2 g sodium bicarbonate, 10 g BSA, 10 µl ITS-X, 5 mL GlutaMAX, 22 mg vitamin C and 10 mL P/S.

- Basal medium S3: 500 ml of MCDB 131 supplemented with 0.72 g glucose, 1.2 g sodium bicarbonate, 10 g BSA, 2.5 ml ITS-X, 5 ml GlutaMAX, 22 mg vitamin C and 10 ml P/S.

- Basal medium S5: 500 ml MCDB 131 supplemented with 2.3 g glucose, 1.47 g sodium bicarbonate, 10 g BSA, 2.5 ml ITS-X, 5 ml GlutaMAX, 22 mg vitamin C, 10 ml P/S and 5 mg heparin (Pharepa 25000UI/5ml, Pharmatex Italy).

- Basal medium S6: 500 ml of CMRL 1066 1X supplemented with 1.1 g sodium bicarbonate, 50 g BSA, 5 ml GlutaMAX, 5 ml P/S, 5 mg heparin, 5.2 ml non-essential MEM amino acids (Corning; 20-025-CI), 84 µg of ZnSO4 (MilliporeSigma ; 10883), 523 µL of trace element A (Corning; 25-021-CI) and 523 µL of trace element B (Corning; 25-022-CI).

# Supplementary Tables

**Supplementary Table 1:** Primary antibodies

| ***Name (Catalogue Number)*** | ***Dilution*** | ***Company*** |
| --- | --- | --- |
|  |  |  |
| Guinea pig anti-Insulin (AB3440) | 1:100 | Chemicon, Millipore**,** Milan, Italy |
| Mouse anti-elastin (MA1-27129) | 1:100 | ThermoFischer, Milan, Italy |
| Mouse anti-Glucagon (G2654) | 1:100 | Sigma-Aldrich, Milan, Italy |
| Mouse anti-Urocortin3 (sc-517449) | 1:50 | Santa Cruz, DBA, Milan, Italy |
| Rabbit anti- Nanog (PC-102) | 1:150 | Kamira Biomedical Company, LiStarFish, Milan, Italy |
| Rabbit anti- Sox2 (MO15040) | 1:200 | Neuromics**,** LiStarFish, Milan, Italy |
| Rabbit anti-cMyc (GTX59578) | 1:500 | GeneTex Inc, LiStarFish, Milan, Italy |
| Rabbit anti-ki67 (TA336568) | 1:150 | Origene Inc., Milan, Italy |
| Rabbit anti-Oct4 (P0873) | 1:400 (1:1000wb) | Sigma-Aldrich, Milan, Italy |
| Mouse anti-Oct4 (sc-5279) | 1:50  (1:500wb) | Santa Cruz, DBA, Milan, Italy |
| Rabbit anti-Somatostatin(sc-13099) | 1:100 | Santa Cruz, DBA, Milan, Italy |
|  |  |  |

**Supplementary Table 2:** Secondary antibodies

| ***Name*** | ***Dilution*** | ***Company*** |
| --- | --- | --- |
|  |  |  |
| Donkey anti-rabbit IgG TRITC (711-025-152) | 1:200 | Jackson Immunoresesrh, LiStarFish, Milan, Italy |
| Donkey anti-rabbit IgG FITC (711-095-152) | 1:200 | Jackson Immunoresesrh, LiStarFish, Milan, Italy |
| Goat anti-mouse IgG FITC (115-095-146) | 1:200 | Jackson Immunoresesrh, LiStarFish, Milan, Italy |
| Goat anti-mouse IgG TRITC (115-025-146) | 1:200 | Jackson Immunoresesrh, LiStarFish, Milan, Italy |
| Goat anti-guinea pig IgG FITC (106-095-003) | 1:100 | Jackson Immunoresesrh, LiStarFish, Milan, Italy |
|  |  |  |

**Supplementary Table 3** : Primers used in qPCR. Primers were designed using the sequences from GenBank (htpp://www.ncbi.nlm.nih.gov/Genbank).

| ***Gene*** | ***T °C Annealing*** | ***Forward*** | ***Reverse*** | ***Expected length bp*** |
| --- | --- | --- | --- | --- |
| CXCR4 | 60 | 5’tgctgtatgtctcgtggtag3’ | 5’ctgaaatcaacccactcctg3’ | 241 |
| E-CAD | 60 | 5’cgtacacagccctaatcatag3’ | 5’gctttggattcctctcacag3’ | 147 |
| FOXA2 | 60 | 5’atgcactcggcttccagtat3’ | 5’taggtgttcatgccgttcat3’ | 146 |
| GAPDH | 60 | 5’ggaaggtgaaggtcggag3’ | 5’gaaatcccatcaccatcttcc3’ | 228 |
| GCK | 60 | 5’cttcaccttctcctttcctg3’ | 5’tcattcaccattgccacc3’ | 174 |
| Glucagon | 60 | 5’ctgtcccttcaagacacagaggag3’ | 5’tccctggcggcaagattatc3’ | 237 |
| GLUT2 | 60 | 5’ttggtgtgatcaatgcacct3’ | 5’gctgccacagtctctcctc3’ | 182 |
| HLAG5 | 60 | 5'ctcaccttcacctcctttccca3' | 5'caatctgagctcttctttctccaca3' | 144 |
| HNF6 | 60 | 5’agaacatgggaaggatagagg3’ | 5’gaagatgagttgcctgaattg3’ | 246 |
| HPRT1 | 65 | 5’atgctgaggatttggaaagggtg3’ | 5’cagagggctacaatgtgatggc3’ | 115 |
| ICA512 | 60 | 5’ggaaggtgaacaagtgctac3’ | 5’cagcgatgtcaatctccttc3’ | 135 |
| Insulin | 65 | 5’ggccatcaagcagatcactgtcct3’ | 5’tgggtcaggtccccagaggg3’ | 93 |
| ISL1 | 60 | 5’aaacaggagctccagcaaaa3’ | 5’ccaagagacccaggatttca3’ | 142 |
| N-CAD | 60 | 5’-ggaccgagaatcaccaaatg-3’ | 5’-cttgaggtaacacttgaggg-3’ | 149 |
| NEUROD | 60 | 5’acagctcccatgtcttccac3’ | 5’aagattgatccgtggctttg3’ | 250 |
| NGN3 | 60 | 5’ccccattctctcttcttttctc3’ | 5’aggtcacttcgtcttccg3’ | 242 |
| NKX2.2 | 60 | 5’tctgaaccttgggagaggc3’ | 5’ggtcattttggcaacaatcacc3’ | 269 |
| NKX6.1 | 60 | 5’acgggaagagaaaacacacg3’ | 5’tggcatccagaggcttattg3’ | 236 |
| PAX4 | 60 | 5’gagggtctggttttccaaca3’ | 5’tgctgtgcagagcgtattcc3’ | 152 |
| PAX6 | 60 | 5’gaatgggcggagttatgatac3’ | 5’gacatatcaggttcacttccg3’ | 395 |
| PDX1 | 60 | 5’cctttcccatggatgaagtc3’ | 5’ttgtcctcctcctttttcca3’ | 269 |
| Somatostatin | 60 | 5’gatccgcgcctagagtttga3’ | 5’agtacttggccagttcctgc3’ | 210 |
| SOX17 | 60 | 5’agcagaatccagacctgcac3’ | 5’ttgtagttggggtggtcctg3’ | 145 |
| UCN3 | 60 | 5’cccacaagttctacaaagcca3’ | 5’tcccgaagaggcgtctctg3’ | 143 |

# Supplementary: Analysis ex vivo

In the various planned experiments, peritoneal washes were carried out at the end of the short (24 h-48 h) or very long term (42 days) post-transplantation periodic sampling in order to detect the different cells of the immune system.

After anaesthetizing the mouse, using a scissors and forceps the outer skin was cut on the peritoneum and was gently pulled it back to exposure the inner skin lining the peritoneal cavity. Then, 4 ml of NaCl 0.9% were injected into peritoneal cavity using a 27g needle, gently massage the peritoneum to dislodge any attached cells into the saline solution. As much fluid as possible was collected and the collected cell suspension was deposited in tubes kept on ice. The collected cell suspension was spun at 1500 rpm for 8 minutes, the supernatant was discarded and the cells was resuspended in desired PBS 1X for counting.

Upon 24 h of graft, peritoneal lavage showed –for both groups (uncoated and coated)- not specific reactivity compatible with the transplant procedure itself (cellularity was mainly comprised of neutrophils). At 48 h of graft, uncoated hiPSC were associated with massive infiltration with macrophages and lymphocytes, while neutrophils percentage was lower. For coated group, at 7 days no lymphocytic activated rosettes were detected; at 21 and 42 days after peritoneal lavage, low degree of inflammation was detected. Individual differences were observed.


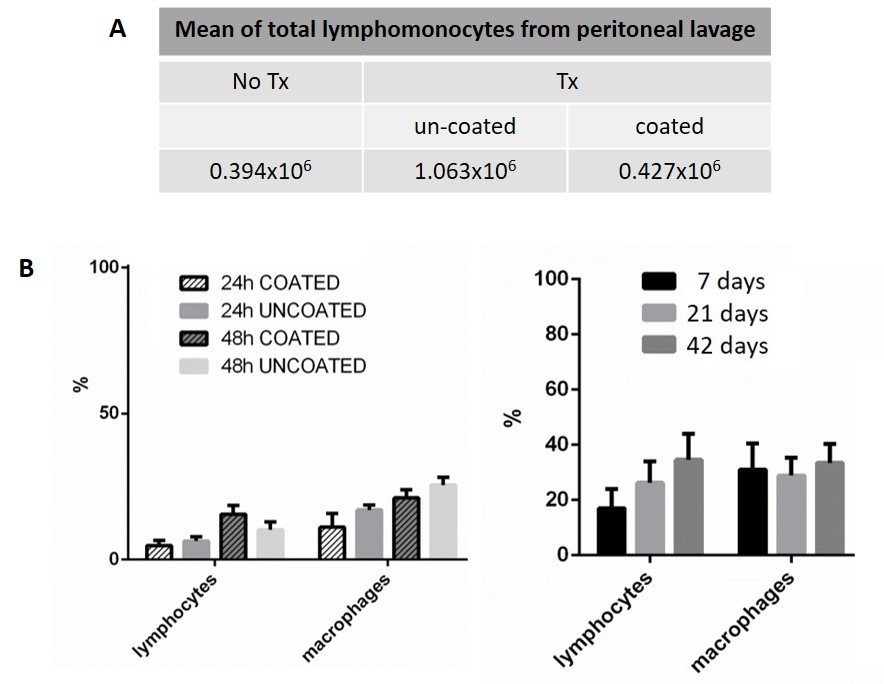


**Supplementary Figure 1**: A) Mean of total lymphomonocytes from peritoneal lavage at 7 days; B) Percentage of the lymphomonocyte populations present in peritoneal lavage.


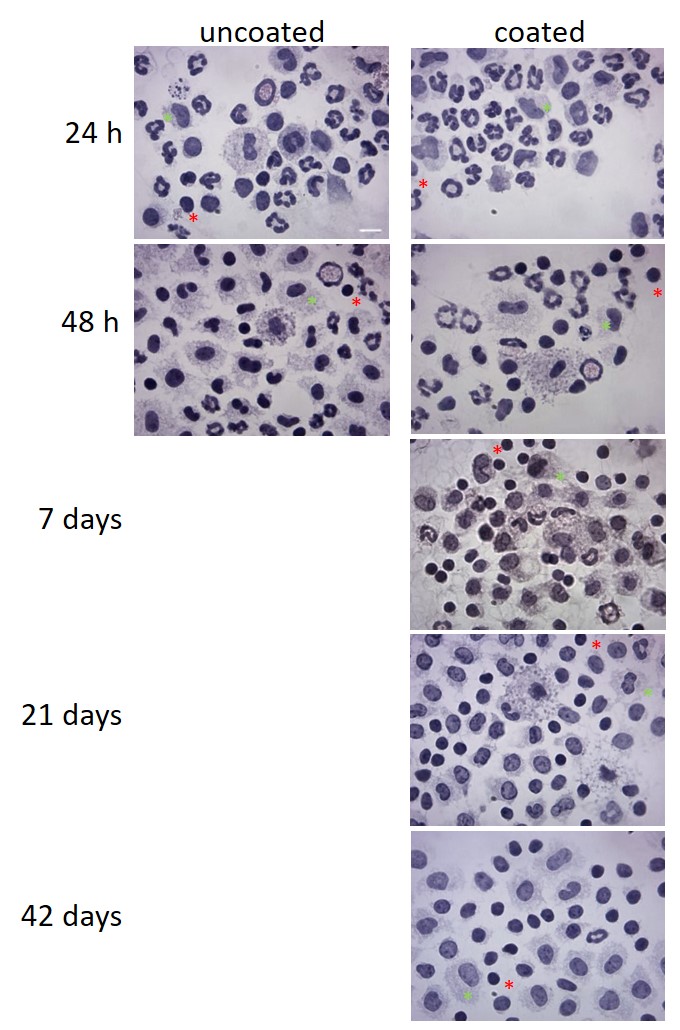


**Supplementary Figure 2**: Hematoxylin/Eosin staining of collected cells from peritoneal lavage at different time periods (100X) (*lymphocytes, *macrophages).

**
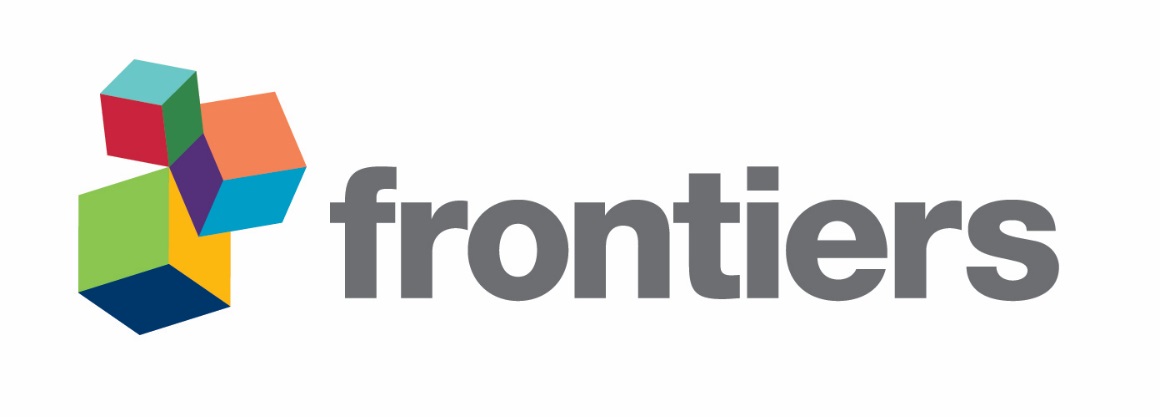
**
